# Comprehensive Combined Proteomics and Genomics Analysis Identifies Prognostic Related Transcription Factors in Breast Cancer and Explores the Role of DMAP1 in Breast Cancer

**DOI:** 10.3390/jpm11111068

**Published:** 2021-10-23

**Authors:** Xuan Li, Hefen Sun, Yifeng Hou, Wei Jin

**Affiliations:** 1 Department of Breast Surgery, Key Laboratory of Breast Cancer in Shanghai, Fudan University Shanghai Cancer Center, Shanghai 200032, China; 19111230032@fudan.edu.cn (X.L.); hfsun14@fudan.edu.cn (H.S.); yihou@fudan.edu.cn (Y.H.); 2 Department of Oncology, Shanghai Medical College, Fudan University, Shanghai 200032, China

**Keywords:** transcription factors, ATAC, alternative splicing, alternative splicing, DMAP1, prognosis, proteomic analysis, breast cancer

## Abstract

Transcription factors (TFs) are important for regulating gene transcription and are the hallmark of many cancers. The identification of breast cancer TFs will help in developing new diagnostic and individualized cancer treatment tools. In this study, we used quantitative proteomic analyses of nuclear proteins and massive transcriptome data to identify enriched potential TFs and explore the possible role of the transcription factor DMAP1 in breast cancer. We identified 13 prognostic-related TFs and constructed their regulated genes, alternative splicing (AS) events, and splicing factor (SF) regulation networks. DMAP1 was reported less in breast cancer. The expression of DMAP1 decreased in breast cancer tumors compared with normal tissues. The poor prognosis of patients with low DMAP1 expression may relate to the activated PI3K/Akt signaling pathway, as well as other cancer-relevant pathways. This may be due to the low methylation and high expression of these pathway genes and the fact that such patients show more sensitivity to some PI3K/Akt signaling pathway inhibitors. The high expression of DMAP1 was correlated with low immune cell infiltration, and the response to immune checkpoint inhibitor treatment in patients with high DMAP1 expression was low. Our study identifies some transcription factors that are significant for breast cancer progression, which can be used as potential personalized prognostic markers in the future.

## 1. Introduction

Breast cancer has the highest incidence rate among females in the US [1]. In women, breast cancer has relatively better prognosis than other cancers, but due to its high incidence rate, breast cancer is still the second leading cause of death from cancer among women. Survival is better for early stage patients, as the overall 5-year survival rate was over 90% for TNM Stage I and II patients, as classified by the American Joint Committee on Cancer (AJCC) Cancer Staging System [2]. Metastasis to distant organs is the major cause of death and is a huge threat for breast cancer patients [2,3]. Exploring the mechanism of malignant transformation and progression of breast cancer is helpful for early diagnosis and treatment.

The progress of cancer mainly occurs by gene regulation, and transcription factors (TFs) are the most studied gene regulatory mechanisms and are the hallmark of many cancers [4,5,6]. Tumor specific genes are strongly influenced by TFs regulation, and abnormal TFs could induce or activate oncogenic signaling or inhibit cancer suppression signaling. Many studies have focused on TFs as cancer driver genes and potential prognostic markers for tumors. Single transcription factors such as estrogen receptors (ER) are steroid hormone-dependent transcription factors in breast cancer and influence the development, growth, and endocrine treatment response of the majority of breast carcinomas [7,8]. As more and more TFs have been discovered, the joint analysis of TFs has received more attention. The epithelial–mesenchymal transition (EMT) pathway has been found to be activated in many carcinoma cells and executes the key step of metastasis [9,10]. Some nucleolar EMT-inducing transcription factors (EMT-TFs), such as SLUG, SNAIL1, TWIST1 and ZEB1, have been studied together and used as biomarkers for EMT as well as for tumor prognosis in breast cancer [11,12]. It is important to explore more specific TFs for complex tumor mechanisms, as this will lay the groundwork for developing new diagnostic and individualized cancer treatment tools, though it remains a challenge.

In recent years, thanks to the development of in-depth sequencing technology in genomics, transcriptomics and proteomics, more and more cancer specific and individualized gene markers have been identified [13]. With the ability to access and explore the data from large non-profit datasets such as TCGA (The Cancer Genome Atlas) and data repositories like GEO (Gene Expression Omnibus), a large number of patients’ gene transcript expression data have been made available to researchers [13,14]. Gene transcription is usually regulated by TFs through binding to specific DNA regions, which are called open chromatin regions. Many experimental approaches have been taken to study open chromatin regions, including CHIP-seq, DNase-seq, MNase-seq, ATAC-seq, etc. [15,16]. Among these, ATAC-seq, which was developed by William J. Greenleaf and Howard Y. Chang, has been applied to a large number of samples in the TCGA database [17,18]. A combined analysis of TFs and open chromatin regions could not only reveal the regulatory differences of TFs but also provide information to researchers to help them find possible TFs through the motif of transcription initiation. Alternative splicing refers to removing introns and keeping exons that lead the precursor mRNA to form the mature mRNA. This is the main mechanism for maintaining protein diversity [19]. Proteins act as the functional executor of genes, and quantitative proteomics using methods such as iTRAQ labeling can provide a great deal of biological information at the post-transcriptional levels. The combined application of a variety of research methods can explore more reliable transcription factors.

MDA-MB-231 HM and MDA-MB-231 Bo cells are highly metastatic potential cells derived from their parental cell, MDA-MB-231 [20]. By comparing the nuclear protein expression between both MDA-MB-231 HM and MDA-MB-231 Bo and MDA-MB-231 cell lines can provide an effective method to identify prognostic-associated TFs in breast cancer. In this study, the overlapped differentially expressed TFs were selected by using the iTRAQ-nano-HPLC-MS/MS methods. Transcriptomic comparisons of these differential genes of these TFs were undertaken in a large cohort of databases to identify the most prognosis related TFs. ATAC-seq data and alternative splicing data from TCGA were applied to construct the TFs regulated network in breast cancer. We also focused on the transcription factor DMAP1 because research into this gene in breast cancer is scarce. In this study, we aimed to find meaningful TFs and their regulation network, which could provide some new ideas for future research into breast cancer.

## 2. Materials and Methods

### 2.1. Cell Culture and Breast Cancer Specimens

MDA-MB-231, HS-578T and BT-474 were bought from ATCC (American Type Culture Collection). SK-BR-3 and T-47D were purchased from the Cell Bank of Type Culture Collection of the Chinese Academy of Science (Shanghai, China). The MDA-MB-231 HM line was developed from the parental MDA-MB-231 cells via four cycles of mice tail vein injections in our laboratory (patent number: 200910174455.4) [21,22] and MDA MB-231 Bo was kindly provided by Dr. Toshiyuki Yoneda (The University of Texas, Houston, TX, USA); both of these exhibited enhanced lung or bone metastasis capacity. All cells were cultured in an appropriate medium as recommended under conditions of 37 °C, a 5% CO_2_ atmosphere, and suitable saturated humidity.

Nineteen pairs of breast carcinomas and paracancerous tissues were randomly collected from patients who underwent surgical treatment for breast cancer at the Fudan University Shanghai Cancer Center. The use of all clinical samples was approved by the Ethics Committee of the Cancer Center of Fudan University.

### 2.2. Nuclear Extraction and iTRAQ-nano-HPLC-MS/MS Analyses

We extracted the nuclei from MDA-MB-231, MDA-MB-231 HM and MDA- MB-231 Bo cell lines by following the instructions of the Nuclear and Cytoplasmic Protein Extraction Kit (Beyotime, P0028; Shanghai, China). The nuclei were subjected to iTRAQ-nano-HPLC-MS/MS analysis. The methods used for the iTRAQ-nano-HPLC-MS/MS analysis was introduced in our previous study [20].

### 2.3. RNA Extraction and Quantitative Real-Time PCR

The total RNA of cell lines was extracted by using the Trizol reagent (Life Technologies; Cat. No. 15596018; Carlsbad, CA, USA). RNA from breast carcinomas and paraneoplastic tissues were extracted by using the AllPrep DNA/RNA/Protein Mini Kit (QIAGEN; Cat. No. 80004; Hilden, Germany) for later PCR analysis. The total RNA of the cell lines and tissues were immediately reverse transcribed to cDNA using the PrimeScript RT Reagent Kit (Perfect Real-Time, Beijing, China; TaKaRa Biotechnology; RR037A; Dalian, China). The subsequent real- time polymerase chain reaction (PCR) was performed with SYBR Premix Ex Taq (TaKaRa Bio; Cat. No. RR820A; Dalian, China) in an ABI Prism 7900 instrument (Applied Biosystems; ABI QuantStudio 6 Flex; Waltham, MA, USA).

DMAP1: F 5′- GCACCGGGAAGTCTATGCC-3′,
DMAP1: R 5′- CACTGTACGGTATCCCTGGC-3′
GAPDH: F 5′-GGAGCGAGATCCCTCCAAAAT-3′
GAPDH: R 5′-GGCTGTTGTCATACTTCTCATGG-3′

### 2.4. Selection of Potential Transcription Factors (TFs)

The TFs list was downloaded from (http://www.tfcheckpoint.org/; accessed on 1 August 2021), and the TFs that had consistently high or low expression in MDA-MB-231 HM and MDA- MB-231 Bo compared with MDA-MB-231 in the results of the iTRAQ-nano-HPLC-MS/MS analysis were chosen as candidate TFs and used for further analysis.

The RNA-sequence data and the corresponding survival and clinicopathological information of TCGA breast cancer patients were downloaded from the TCGA data portal (https://tcga-data.nci.nih.gov/tcga/; accessed on 1 August 2021). Other datasets (GSE20685, GSE9014, GSE39004, GSE20712 and GSE150576) for verifying the results were downloaded from the Gene Expression Omnibus (GEO) repository (https://www.ncbi.nlm.nih.gov/geo/; accessed on 1 August 2021).

### 2.5. The ATAC-seq Analysis

The ATAC-seq (Assay for Transposase-Accessible Chromatin with high throughput sequencing) data of TCGA-BRCA were taken from the research of Corces et al. [18] (https://gdc.cancer.gov/about-data/publications/ATACseq-AWG; accessed on 1 August 2021). The chromosome sites of differential accessibility peaks (DAPs) were annotated by TxDb.Hsapiens.UCSC.hg38.knownGene and org.Hs.eg.db, which include exon, promoter, 3′UTR, 5′UTR, intron, distal intergenic, and downstream regions. The peaks corresponding to gene names were annotated by the ChIPseeker package. Peaks that correlated with TFs were based on Pearson’ correlation analysis, and an absolute Pearson value greater than 0.5 and a *p*-value less than 0.05 were considered to be statistically significant. The relationships between the multiple interactive datasets were demonstrated by the UpSet package in R software, and the biological function enrichment of DAPs located near the upstream and downstream of the transcription start site (TSS) was performed by the clusterProfiler package in R software.

### 2.6. Identification of Survival Associated AS Events

AS events can be classified into exclusive exon (ME), alternate donor (AD), alternate acceptor (AA), alternate promoter (AP), retained intron (RI), exon skip (ES) and alternate terminator (AT) events. The percent spliced index (PSI) values for each AS events of the breast cancer samples were downloaded from the TCGAsplice-Seq database (https://bioinformatics. mdanderson.org/TCGASpliceSeq; accessed on 1 August 2021) [23].

Upset plots were adopted to visualize the TFs and their corresponding ATAC core peak genes were related to AS events by using UpSetR package (R version 4.0.3). AS events with *p* < 0.05 in univariate Cox regression analysis for DFS were selected for further research and were displayed by upset plots.

### 2.7. Construction of the TFs-ATAC-SF-AS Regulatory Network

A list of 119 splicing factors (SFs) was obtained from the study of Seiler et al. [24]. The gene expression information of the SFs was taken from the TCGA RNA-Seq database. To estimate the correlation between the SFs and survival-related AS events, a correlation analysis was executed by using Pearson correlation analysis. Pearson’s correlation coefficients greater than 0.5, and *p*-values less than 0.05 were considered to be statistically significant and indicates a strong correlation.

The potential TFs-ATAC-SF-AS regulatory network was constructed by the previous analyses and visualized by Cytoscape software (version 3.7.2).

### 2.8. DMAP1 Expression and Survival Analysis

The expression levels of DMAP1 in breast cancer, tumor-adjacent tissues and normal tissues were analyzed by Breast Cancer Gene-Expression Miner v4, along with the expression according to different molecular subtypes of breast cancer (http://bcgenex.ico.unicancer.fr/BC-GEM/GEM-requete.php; accessed on 1 August 2021). The survival prognostics of DMAP1 in breast cancer were analyzed with Kaplan-Meier Plotter (https://kmplot.com/analysis/; accessed on 1 August 2021).

### 2.9. Bio-Informatic Analysis of DMAP1

The selected differentially expressed or methylated genes between groups with high and low DMAP1 expression were analyzed using the “limma” package in R software with a *p*-value less than 0.05 and a log |fold change| > 2. Gene enrichment analysis used Kyoto Encyclopedia of Genes and Genomes (KEGG) pathway analysis (https://david.abcc.ncifcrf.gov; accessed on 1 August 2021).

### 2.10. Analysis of DMAP1 Expression and Immune Features

Single-sample gene set enrichment analysis (ssGSEA) was used on the GEO datasets to analyze the relationship between the immune features and DMAP1 expression. Relationships between the tumor-infiltrating lymphocytes (TILs) and the expression of DMAP1 in pan cancer were obtained from TISIDB (http://cis.hku.hk/TISIDB; accessed on 1 August 2021).

The predicted responses to immune-checkpoint inhibitor (ICI) therapy were obtained from the TIDE website (http://tide.dfci.harvard.edu/; accessed on 1 August 2021) and their relationships with DMAP1 were analyzed by an unpaired *t*-test.

### 2.11. Exploring the Correlations of Drug Interaction and Sensitivity with DMAP1

The relationships of DMAP1 expression were explored with chemicals obtained from the Comparative Toxicogenomics Database (CTD) (http://ctdbase.org/; accessed on 1 August 2021), which is a free online database that provides information including chemical–gene/protein interactions. The results were visualized using Cytoscape (Version 3.7.1).

The drug IC50 data of breast cancer cell lines and the gene expression data of these cell lines were obtained from the Genomics of Drug Sensitivity in Cancer database (GDSC) (https://www.cancerrxgene.org/; accessed on 1 August 2021). The relationships of DMAP1 to the drug IC50 values were analyzed using the Pearson correlation coefficient index in SPSS.

### 2.12. Statistical Analyses

In this study, R software (version 3.6.1), SPSS (version 25), and Parism 8 were the primary software packages used. A two-tailed *p*-value of less than 0.05 was used to judge statistical significance in our analyses.

## 3. Results

### 3.1. Workflow of This Study

**Figure 1:**
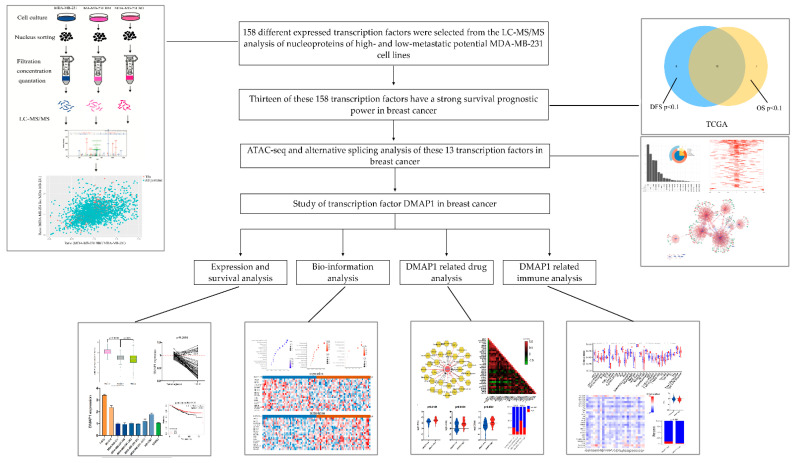
Flow chart of the whole study design.

The workflow of this study is shown in Figure 1. We first purified the nuclei of MDA-MB-231 cells and their derived cell lines, MDA-MB-231 HM and MDA-MB-231 Bo. The nucleus proteins were identified by iTRAQ-nano-HPLC-MS/MS analysis, and 158 transcription factors (TFs) showed consistent high or low expression trends when MDA-MB-231 HM and MDA-MB-231 Bo were compared with MDA-MB-231 cells. In the TCGA breast cancer cohort, 20 genes among these 158 TFs showed a statistically significant difference in OS, while 21 genes showed a statistically significant difference in DFS. Thirteen genes showed prognostic ability for both OS and DFS in breast cancer.

### 3.2. The Transcription Regulation Core Peaks of the 13 TFs in Breast Cancer

**Figure 2:**
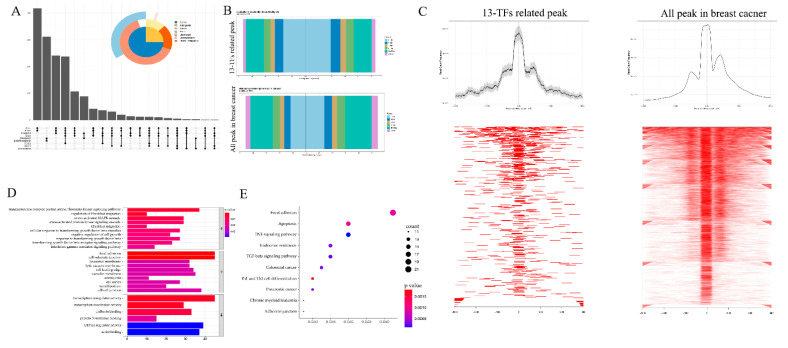
Correlation peaks of the 13 transcription factors. (**A**) Upset plots showing the distribution of all peaks that are related to the 13 TFs. (**B**) Distribution of the 13 TFs and breast cancer related peaks at the transcription start sites (TSSs). (**C**) Visualization of the intensity of the 13 TFs related peaks and all breast cancer peaks on both sides of the TSSs. (**D**,**E**) GO and KEGG enrichment analysis of the 13 TFs related peaks.

To explore the transcription regulation information of these 13 TFs in breast cancer, we analyzed these 13 TFs’ chromatin accessibility with the ATAC-seq data of TCGA-BRCA. Peaks with a correlation rate higher than 0.6 with the targeted TFs and a *p*-value less than 0.05 were selected as the core peaks for these 13 TFs in breast cancer. The distribution of all these peaks is shown in Figure 2A. Most of the peaks were distributed in genic, intron or promoter regions, while a few peaks were distributed downstream. Next, we analyzed the core peaks’ distribution near the transcriptional start sites (TSSs). The distribution of core peaks near TSSs can be found in Figure 2B,C. It can be seen that both sides of the binding regions of the TSSs in the range of 0–1 kb are larger and the proportion of peaks upstream of TSS increased, suggesting that the openness of these regions increased and that the ability of binding transcription factors was promoted. These results mean that the genes corresponding to these core peaks conformed with the regulation of the 13 TFs.

To further investigate the potential biological behavior of the TFs and their regulated core peaks, we used the clusterProfiler package to perform GO (Figure 2D) and KEGG (Figure 2E) enrichment analysis on them. The biological processes with significant enrichment of the TFs and their regulated core peaks were mainly in the transcription activity, focal adhesion, and transforming growth factor beta signaling pathways. This enrichment of biological processes was closely related to tumor progression.

### 3.3. The Alternative Splicing (AS) Events of the 13 TFs and Their Regulated Genes

**Figure 3:**
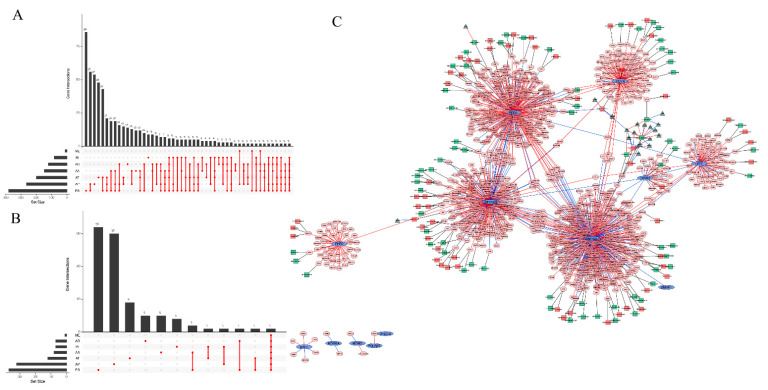
Alternative splicing (AS) events of the 13 TFs and their regulated genes. (**A**) Upset plot of the interactions among seven of the AS events of the 13 TFs and their regulated genes. (**B**) Upset plot of the interactions among seven types of the DFS-related AS events of the 13 TFs and their regulated genes. (**C**) Regulatory network of 13 TFs and their regulatory genes, along with the survival-related AS events of these genes and the splicing factors (SFs) associated with these AS events in breast cancer. The blue ellipses represent the 13 TFs and the pink octagons represent the genes regulated by the TFs. The red lines between the ellipses and the octagons indicated that the TFs positively regulated the genes, while the blue lines indicated that the TFs negatively regulated the genes. The squares represent AS events, and the red squares mean that the AS events are risk factors for DFS, while the AS events indicated by green squares are favorable factors for DFS. The dark green triangles attached to the squares are SFs, and the red lines between the triangles and the squares indicate that the SFs positively regulated the AS events, while the blue lines mean that the SFs negatively regulated the AS events.

To explore the AS events of TFs and their related genes, we analyzed the TCGASpliceSeq data and found 955 AS events in total. The exon skipping (ES) events were the most frequent events, while the exclusive exon (ME) events were the least frequent (Figure 3A). Univariate Cox analysis was applied to explore the potential relationships between these AS events and DFS in breast cancer. In total, 127 DFS-related AS events were identified (Figure 3B). These DFS-related AS events may be modified by certain splicing factors (SFs), as SFs are RNA binding proteins that can recognize regulatory elements in pre-mRNA. We explored the potential association between AS events and SFs in the TCGA-BRCA data set and found that some SFs can regulate several AS events simultaneously and that one AS event can also be controlled by many SFs (Figure 3C, Table S1). We constructed a network of 13 TFs and their regulated genes, along with these genes’ survival-related AS events and the AS event correlated SFs. The network vividly visualizes the important TFs regulation data in breast cancer.

### 3.4. DMAP1 May Play a Cancer Suppressive Role in Breast Cancer

After checking previous research, we found that most of these 13 TFs were well studied in breast cancer as effective transcription factors. This also demonstrates the accuracy of our search method for finding the TFs that were associated with breast cancer. However, among these 13 TFs, we noticed that the transcription factor DMAP1 has seldom been reported in breast cancer. We next explored the clinical and bio-functional effect of the transcription factor DMAP1 in breast cancer.

**Figure 4:**
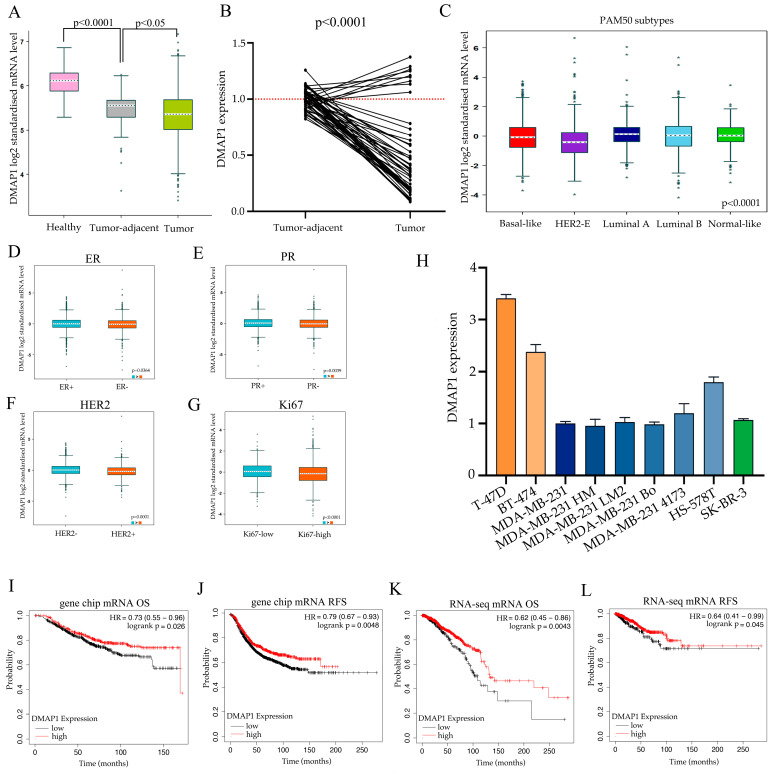
DMAP1 plays a tumor-suppressive role in breast cancer. (**A**) The expression of DMAP1 in breast cancer, tumor-adjacent tissues, and normal tissues from the GTEx and TCGA dataset. (**B**) The gene expression of DMAP1 in 19 pairs of matched breast carcinomas and tumor adjacent tissues. (**C**) DMAP1 expression according to the PAM50 subtypes in breast cancer. (**D**–**G**) DMAP1 expression in different ER, PR, HER2, and Ki67 classifications. (**H**) DMAP1 expression in breast cancer cell lines from our laboratory. (**I**,**J**) Kaplan-Meier (K-M) curves showing the overall survival (OS) and recurrence-free survival (RFS) associated with high and low expression of DMAP1 from the gene chip results in a collection of GEO datasets. (**K**,**L**) Kaplan-Meier (K-M) curves showing the OS and RFS associated with high and low expression of DMAP1 from RNA-seq results in the TCGA dataset. * the data of a sample.

We first explored the expression of DMAP1 in breast cancer, tumor adjacent tissues, and normal tissues in the GTEx and TCGA datasets. The mRNA levels showed that the expression of DMAP1 was significantly the highest in normal tissues, second in tumor adjacent tissues, and lowest in tumor tissues (Figure 4A). We next used PCR to analyze the expression of DMAP1 in 19 pairs of matched carcinomas and tumor adjacent tissues from surgical patients at our hospital and found that DMAP1 was expressed significantly lower in carcinoma tissues than in paracarcinomas tissues (*p* < 0.0001, Figure 4B). The PAM50 subtypes of breast cancer showed that the DMAP1 expression was higher in luminal subtypes than in the basal-like and HER2-amplified subtypes (Figure 4C). The correlation of DMAP1 and molecular typing markers for breast cancer showed that DMAP1 expression was lower in ER negative (Figure 4D), PR negative (Figure 4E), and HER2 positive subtypes (Figure 4F), as well as in tumors with high Ki67 expressed (Figure 4G). We used cell lines from our laboratory to explore the DMAP1 gene expression patterns and found that its expression was higher in luminal cell lines such as T-47D and BT-474 than in triple negative or HER2 positive cell lines (Figure 4H). DMAP1 also showed survival predictive value for breast cancer patients. As shown in Figure 4I–L, elevated expression levels of DMAP1 were significantly associated with higher overall survival and relapse free survival, according to the results of mRNA microarrays and RNA-seq datasets separately. All these findings indicate that DMAP1 may be used as a tumor suppressor in breast cancer.

### 3.5. Identification of Differentially Expressed Genes (DEGs) between the Group with High and Low DMAP1 Expression

**Figure 5:**
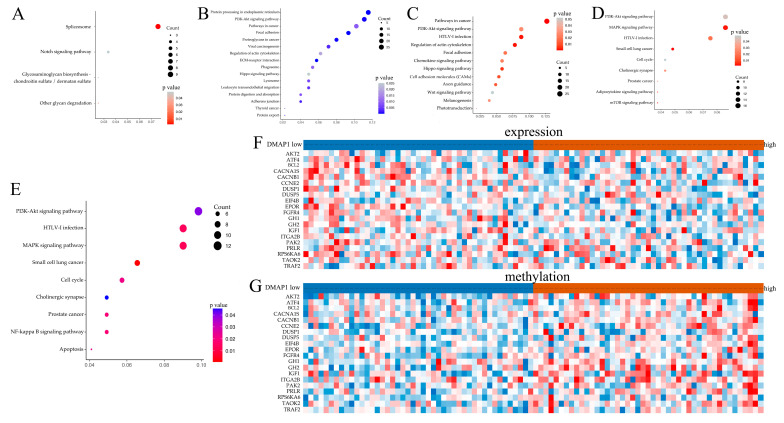
Bioinformatics analysis of DMAP1. (**A**) Bubble plots showing the KEGG enrichment analysis of the highly expressed genes in the high DMAP1 expression group. (**B**) Bubble plots showing the KEGG enrichment analysis of the low expression genes in the high DMAP1expression group. (**C**) Bubble plots showing the KEGG enrichment analysis of the genes with a high methylation level between the high DMAP1 expression group and the low DMAP1 expression group in the GSE39004 dataset. (**D**) Bubble plots showing the KEGG enrichment analysis of the genes with a high methylation level between the high DMAP1 expression group and the low DMAP1 expression group in the GSE20712 dataset. (**E**) Bubble plots showing the KEGG enrichment analysis of the genes with simultaneously high methylation and low expression between the high DMAP1 expression group and the low DMAP1 expression group in the GSE20712 dataset. (**F**,**G**) Heatmaps of the expression and methylation level of genes in the PI3K-Akt signaling pathway between the group with high and low DMAP1 expression.

The RNA-seq data from TCGA were analyzed using the limma package in R software (|logFC| > 2, adjusted *p*-value < 0.05) to find DEGs between the groups with high and low DMAP1 expression, and 1531 upregulated and 1581 downregulated genes were identified in the high DMAP1 expression group compared with the low DMAP1 expression group. KEGG enrichment analysis was applied to better understand the bio-function of DMAP1 using the DEGs. The KEGG pathway analysis showed that the highly expressed DEGs were enriched in the spliceosome, the notch signaling pathway, etc. (Figure 5A), while the low expression DEGs were enriched in the PI3K-Akt signaling pathway, pathway in cancer, focal adhesion, hippo signaling pathway, etc. (Figure 5B). These pathway analyses indicated that low DMAP1 expression may be involved in cancer progression.

### 3.6. Genes Methylation and Expression Combined Analysis between Groups with Low and High DMAP1 Expression

As DMAP1 is involved in DNA methylation, we suspect that it may influence the methylation of some genes and then their expression to play its inhibitory role in breast cancer. We used methylation profiling in an array of two independent datasets (GSE39004 and GSE20712) to explore the differential methylation levels of genes between groups with high and low DMAP1 expression. According to the KEGG analysis, genes with high methylation levels in the high DMAP1 expression group were enriched in the PI3K-Akt signaling pathway, as well as some cancer related pathways in both cohorts (Figure 5C,D). To further verify the relationship between gene expression and methylation in the high DMAP1 expression group, we selected genes with high methylation and low expression levels in the high DMAP1 expression group from the GSE20712 dataset. KEGG enrichment analysis also confirmed that in the high DMAP1 expression group, the genes in the PI3K-Akt signaling pathway, the MAPK signaling pathway, and some cancer related pathways had high methylation but low expression (Figure 5E). Heat maps were used to exhibit the expression levels (Figure 5F) and the methylation levels (Figure 5G) of genes in the PI3K-Akt signaling pathway between the groups with high and low DMAP1 expression.

### 3.7. Drug Regulation and Drug Sensitivity of DMAP1

**Figure 6:**
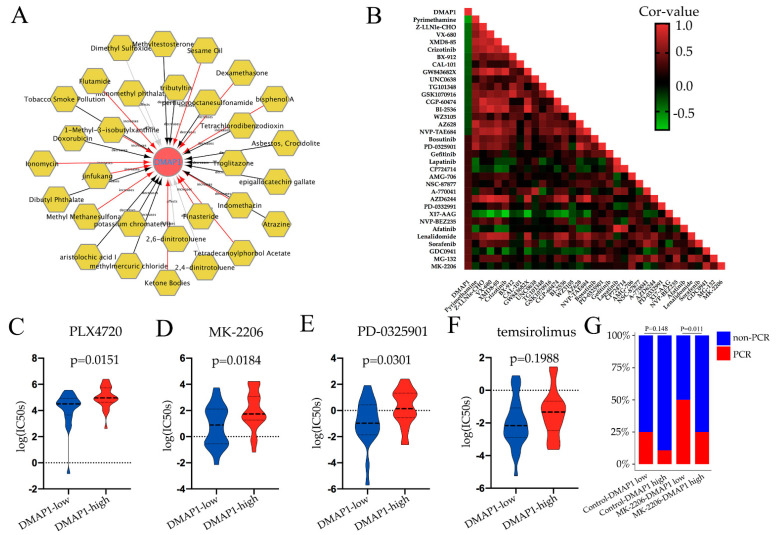
Drug sensitivity analysis of DMAP1. (**A**) The relationships of drugs or chemicals with DMAP1 expression, taken from the Comparative Toxicogenomics Database (CTD). The black arrows indicate that the compound could decrease DMAP1 expression, the red arrows indicate that the compound could increase DMAP1 expression, and the gray arrows indicate that the influence of the compound on the DMAP1 expression was unclear. (**B**) The correlation analysis of DMAP1 expression and drug IC50 values in breast cancer cell lines from the GDSC dataset. The color of each cell in the heat map corresponds to the Pearson correlation coefficient value. (**C**–**F**) Violin plots exhibiting the IC50 data of some PI3K/AKT/mTOR pathway inhibitors (PLX4720, MK-2206, PD-0325901, and temsirolimus) in the group with low and high DMAP1 expression. (**G**) The PCR rate in the group with low and high DMAP1 expression in different arms of the I-SPY 2 trial.

To investigate the potential drugs or chemicals that may modulate the expression of DMAP1, we searched the Comparative Toxicogenomics Database (CTD) and constructed a gene–chemical network using Cytoscape (Figure 6A). The network showed that some commonly used chemotherapy agents for breast cancer could affect DMAP1 gene expression. For example, doxorubicin was found to decrease the expression of DMAP1, while dexamethasone could increase its expression. Because of the small amount of research data available, we could not draw conclusions on the effect of chemotherapy drugs on DMAP1 expression, but we found that many carcinogenic substances, such as aristolochic acid I, asbestos, atrazine, and tobacco smoke pollution, can lead to a decrease in DMAP1 expression. A heatmap was used to show the correlations between DMAP1 expression and the IC50 of some drugs in breast cancer cell lines (Figure 6B). The IC50 of many inhibitors that belong to the PI3K/AKT/mTOR pathway showed a positive correlation with DMAP1 expression. A violin plot was used to exhibit some of the IC50 data of the PI3K/AKT/mTOR pathway inhibitors in the group with low and high DMAP1 expression, and the low DMAP1 group had a lower IC50 for these drugs, which means this group of patients may be more sensitive to PI3K/AKT/mTOR pathway inhibitors (Figure 6C–F). We verified these with the results in the I-SPY 2 trial, in which patients were randomly assigned to receive neoadjuvant chemotherapy with paclitaxel plus MK-2206 (an AKT inhibitor) or control drugs (without MK-2206). From the stacked bars, we could see that the low DMAP1 expression group had a statistically significant higher PCR rate than the high DMAP1 expression group in the MK-2206 treatment arm (Figure 6G).

### 3.8. High DMAP1 Was Correlated with Low Immune Infiltration Cells and May Derive Less Benefit from ICB Treatment

**Figure 7:**
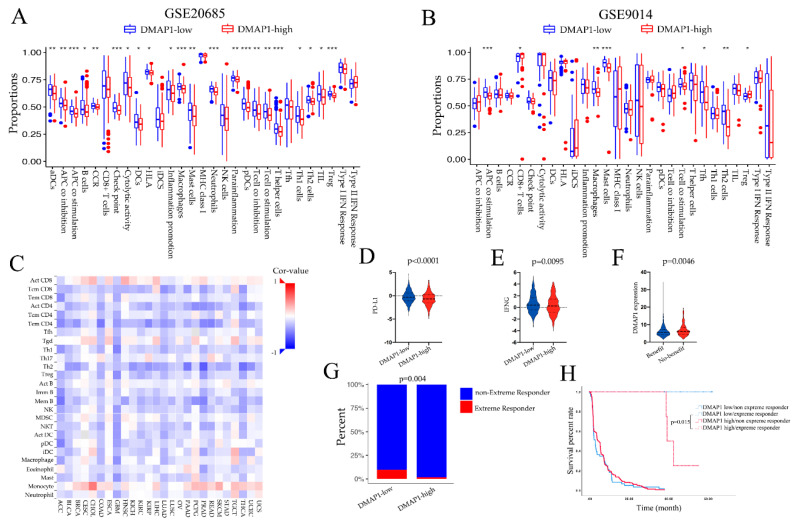
High DMAP1 expression was correlated with a low immune infiltration environment. (**A**,**B**) The ssGSEA scores of immune signatures in the GSE20685 and GSE9014 cohorts according to their DMAP1 classification. (**C**) Pearson correlations of the immune feature scores were obtained from the ssGSEA analysis and the DMAP1 expression of 26 tumor types from the TCGA dataset. The expression levels of PD-L1 (**D**) and IFNG (**E**) were examined according to the DMAP1 classification. (**F**) The expression of DMAP1 in patients with and without benefit from immune checkpoint inhibitor therapy identified through TIDE analysis. (**G**) The extreme response rate in the low and high DMAP1 groups in advanced clear cell renal cell carcinoma patients who received PD-1 blockade therapy (from the study of Braun et al. [25]). (**H**) Kaplan-Meier (K-M) curves showing the survival of patients classified according to their extreme response status and the DMAP1 expression from the study of Braun et al. Note: * means *p* < 0.05; ** means *p* < 0.01; *** means *p* < 0.0001.

To examine whether the DMAP1 expression was correlated with immune features, we first applied ssGSEA analysis in GSE20685 and GSE9014. The high DMAP1 expression group showed significant low enrichment in most immune features such as macrophages, T cell co-stimulation, TIL, NK cells, etc., in two independent cohorts (Figure 7A,B). As the DMAP1 expression level could identify distinct immune features in breast cancer, we next studied the relationship between tumor-infiltrating lymphocytes (TILs) and the expression of DMAP1 in solid pan-cancers of the TCGA dataset (Figure 7C). The results showed that DMAP1 expression was negatively correlated with most immune features in the majority of solid tumors; these results were consistent with our previous findings in breast cancer.

As we found that high DMAP1 expression was negatively correlated with most immune cell infiltrations and other immune features such as immune checkpoint CD274 (PD-L1) (Figure 7D) and IFN-gamma (Figure 7F), we wanted to find out whether it could predict the response to immunotherapy. The predicted immune checkpoints therapy benefits to patients identified through TIDE analysis had lower DMAP1 expression than those patients who experienced no benefit (Figure 7F). We used data on the response to PD-1 blockade in advanced clear cell renal cell carcinoma to further explore the effect of DMAP1 expression and ICI therapy. We found that the low DMAP1 group had a higher extreme response than the high DMAP1 group (*p* = 0.004) (Figure 7G), and patients in the low DMAP1 expression group with an extreme response had a significantly longer survival rate than patients with an extreme response in the high DMAP1 expression group (*p* = 0.015) (Figure 7H). All these results suggest that patients with low DMAP1 expression may benefit more from ICB treatment.

## 4. Discussion

In this study, we used iTRAQ-nano-HPLC-MS/MS analysis to identify 13 transcription factors (TFs) that were enriched in breast cancer cells with high metastatic capacity. The nearby transcriptional start sites (TSSs) of these TFs were wide, which means that their transcription ability in breast cancer was high. Pathway enrichment analysis of the core peaks of these 13 TFs showed a close relationship to tumor process pathways such as transcription activity, focal adhesion, and transforming growth factor beta signaling pathways. The survival related alternative splicing (AS) events of TFs analysis further expanded the TFs’ transcriptome diversity. In this part of our study, we constructed a comprehensive network of TFs, core peak genes, AS and splicing factors (SFs) in breast cancer that correlated with breast cancer progress.

Transcription factors (TFs) can decode the DNA sequence and directly interpret the genome. TFs control many physiological and pathological processes, such as developmental patterning, cell differentiation, immune response, etc. [4,26]. As the TFs play their transcription role by combining with a particular DNA sequence, their nuclear expression is very critical. In our study, we purified the nuclei from normal breast cancer cells and those with high metastatic potential to find 13 TFs. These TFs also showed prognostic ability for breast cancer, so they may be important for the regulation of breast cancer. Previous studies analyzed some of the TFs that we found in breast cancer. Bromodomain-containing protein 4 (BRD4) is a part of the gene super-enhancer regions’ transcriptional machinery and governs the expression of many genes that are critical for cancer progress [27]. In breast cancer, inhibited BRD4 reduces the epithelial–mesenchymal transition (EMT) by decreasing the expression of the snail gene, which further reduces the migration and invasion of breast cancer cells [28]. In triple negative breast cancer, BRD4 can also promote breast cancer migration and invasion by regulating Jagged1 and Notch1 signaling expression [29]. Luminal breast cancer that received AKT inhibitor therapy can acetylate FOXO3a, which recognizes BRD4 and then recruits it to the CDK6 gene promoter and induces its transcription. BRD4 promotes the transcription of CDK6, inducing resistance to AKT inhibitor therapy [30]. The zinc finger MYND-type containing 8 (ZMYND8) is a well-studied transcription factor and it can be recruited to the damaged DNA site and facilitate homologous recombination [31]. In breast cancer, ZMYND8 was found to interact with HIF-1α and HIF-2α, and it can also recruit of BRD4 and release paused RNA polymerase II to enhance the HIF-induced oncogenicity [32]. These previous analyses of some TFs provide indirect proof that the 13 TFs identified in our study may play an important role in regulating the progression of breast cancer.

As TFs play a role through binding to specific DNA sequences to regulate transcription, determining a TF–DNA binding motif is very important for identifying TF-related genes for further analysis [33]. During gene transcription, some chromatin sites open to allow certain regulatory proteins, including TFs, to bind; this characteristic of chromatin is called chromatin accessibility. In this study, we used ATAC-seq analysis, which is a novel method used to study chromosome accessibility developed by Prof. William Greenleaf [18], to find the most likely transcription regulatory network of the 13 TFs in breast cancer. We found many known or novel genes that may be regulated by the 13 TFs. In our study, we found that the transcription factor BRD4 positively regulated the transcription of Notch3 and verified this result in a study of ovarian cancer. In ovarian cancer, researchers found that knockdown of BRD4 could significantly decrease Notch3 expression, and a chromatin immunoprecipitation test revealed that BRD4 existed in the promoter of Notch3 [34]. The analysis of TFs and its regulated genes can help us better understand the progression of breast cancer at the genetic level.

Post-transcriptional processes by alternative splicing (AS) could make the precursor mRNA into multiple mature mRNA that could expand the diversity of the transcriptome and proteome in a limited genome [35]. Dysfunctional AS leads to many changes in tumor cells that, for example, promote cell proliferation, develop tumor angiogenesis, lead to immune escape, and result in therapy resistance [36,37,38,39]. TFs with different AS variants have different effects on cancer, as they can regulate different classes of genes or regulate classes of genes with different effects [40]. A gene could have many AS events, and different AS events of a particular gene could have disparate prognoses. In our study, RUNX1 showed two alternate terminator (AT) events in breast cancer, and one showed favorable prognosis while another showed unfavorable prognosis. Comprehensive analysis of the TFs coding genes could make bio-informatic research more rigorous and accurate.

DNA Methyltransferase 1 Associated Protein 1 (DMAP1) was among the 13 TFs in our study, and it has been rarely studied in breast cancer, so we focused on it for further analysis. DMAP1 was reported to combine with many other proteins to form distinct complexes involved in regulating transcription. Its function in tumor progression is controversial. Lee and colleagues reported that DMAP1 knockdown in colorectal cancer cells could slow and finally ceased cell growth, which may be because the low DMAP1 reactivated some tumor suppressive genes by reducing their CpGs methylation [41]. In other studies, DMAP1 was found to play a tumor-suppressive gene function. In neuroblastoma, low DMAP1 inhibited through MYCN-related ATM/p53 pathway activation, contributing to tumorigenesis [42]. DMAP1 was reported to be stabilized by MDGA2, and their interaction can activate the key elements of the p53/p21 signaling pathway that play a tumor-suppressive role in gastric cancer [43]. In our study, we found that DMAP1 also had a tumor-suppressive function in breast cancer. As both the online database and our own data show, the expression of DMAP1 was lower in tumor tissues than in non-tumor tissues, and analysis of the large amount of data indicated that low expression of DMAP1 was associated with poor survival outcomes.

The reason why low expression of DMAP1 was correlated with poor prognosis in breast cancer is not clear, but the bioinformatic analysis between high and low expression of DMAP1 revealed some clues. The genes enriched in the low DMAP1 expression group showed pathways in cancer as well as the PI3K/Akt signaling pathway. The PI3K/AKT/mTOR pathway is crucial in breast cancer, as it is involved in tumor proliferation, survival, motility, metabolism and anti-tumor therapy resistance, as well as the immune response, and the activated PI3K/Akt signaling pathway is involved in poor prognosis [44]. DNMT1 is generally considered to maintain DNA methylation patterns, and a previous study found that it can promote methylation of promotor genes that inhibit this gene’s expression then affect pathways like the PI3K/Akt/mTOR signaling pathway [45]. As DMAP1 is also named Dnmt1-associated protein 1, and it has a binding role and a potent activation role in DNMT1 methylation, we speculate that DMAP1 may control the biological properties of breast cancer through its gene methylation ability [41,46]. We explored some databases that co-examined the correspondence between gene expression and methylation in breast cancer tissues. As we detected differences in the methylation level of genes between the group with high and low DMAP1 expression, we found that PI3K/Akt signaling pathway genes with a high level of methylation were enriched in the high DMAP1 expression group. Resetting the methylation patterns may change gene expression, as further combined analysis showed that the PI3K/Akt signaling pathway genes had a low expression level in the high DMAP1 expression group. The low DMAP1 group showed high activation of the PI3K/Akt signaling pathway; thus, blocking the PI3K-AKT pathway may be helpful for these breast cancer patients. We analyzed the relationships between drug IC50 and DMAP1 expression in breast cancer cell lines and found that higher DMAP1 expression had a higher IC50 for some PI3K/Akt signaling pathway inhibitors such as MK-2206. We analyzed these results in a clinical trial that used MK-2206 and a control drug as neoadjuvant chemotherapy. We found that the low DMAP1 group had higher PCR rates, especially in the MK-2206 treatment arms. All these results mean that the patients with low DMAP1 expression may be more sensitive to PI3K/Akt signaling pathway inhibitors.

In our study, we analyzed the relationship between DMAP1 expression and the tumor immune micro-environment. The data shows that the DMAP1 expression was negatively correlated with most infiltration immune cell types, not only in breast cancer but also in other solid tumors, which revealed its universal application in tumors. The tumor immune microenvironment is critical to the ICI treatment response, and researchers have analyzed it in pan-cancer data from the TCGA dataset [47]. Recent research divided triple-negative breast cancer subtypes according to their infiltration immune cells into three clusters: immune-desert, immune-inflamed, and innate immune-inactivated clusters [48]. The immune-desert phenotype tumors rarely responded to anti-PD-1/PD-L1 therapy, while the immune-inflamed tumor type has abundant adaptive and innate immune cell infiltration, and it is predicted to have a significantly better ICI therapy response and good survival prognosis in tumors [48,49,50]. The high infiltration rate of most immune cells in low DMAP1 group, as well as the high rates of PD-L1 and IFN-gamma, leads us to speculate that the response rate to ICI treatments may be different according to DMAP1 expression. As the ICI treatment response rates and gene expression profile data in breast cancer were unobtainable, we verified our speculation in advanced clear cell renal cell carcinoma patients that received ICI treatment. The low DMAP1 expression group had a higher extreme response rate than patients in the high DMAP1 expression group, and the survival prognosis was much better in the extreme response patients in the low DMAP1 group. All these findings indicate that patients with low DMAP1 expression may benefit more from ICI treatment.

## 5. Conclusions

In summary, by using the iTRAQ-nano-HPLC-MS/MS analyses of the proteins from the nucleus of breast cancer cells with high and low metastatic potential, we identified some candidate transcription factors of breast cancer. By combined analysis of the protein expression data from massive publicly available gene expression datasets, ATAC-seq data and SpliceSeq data, we constructed a network of 13 TFs and their regulated genes with those genes’ survival-related AS events and its correlated SFs. The network vividly visualizes the important regulation TFs in breast cancer and could provide some clues for further analysis. Among the 13 TFs in our study, DMAP1 has been the least reported in breast cancer. We used real world and network data to confirm that the expression of DMAP1 decreased in breast cancer tumors compared with normal tissues. The poor prognosis of patients with low DMAP1 expression may be related to the activated PI3K/Akt signaling pathway, as well as other cancer relevant pathways, and this may be because of the low methylation and high expression of genes in this pathway. Patients in the low DMAP1 expression group may be more sensitive to PI3K/Akt signaling pathway inhibitors. High expression of DMAP1 was correlated with low immune cell infiltration, and the response to ICI treatment for patients with high DMAP1 expression was low. Our study has identified a number of transcription factors that are significant for breast cancer progression, which might be used as personalized potential prognostic markers in the future.

## Data Availability

The datasets used and analyzed during the current study are available from the Gene Expression Omnibus (GEO) (https://www.ncbi.nlm.nih.gov/geo/), TCGA data portal (https://tcga-data.nci.nih.gov/tcga/), the ATAC-seq (https://gdc.cancer.gov/about-data/publications/ATACseq-AWG), the TCGAsplice-Seq database (https://bioinformatics.mdanderson.org/TCGASpliceSeq), TISIBD (http://cis.hku.hk/TISIDB), TIDE website (http://tide.dfci.harvard.edu/), the Comparative Toxicogenomics Database (CTD) (http://ctdbase.org/), the Genomics of Drug Sensitivity in Cancer database (GDSC) (https://www.cancerrxgene.org/), and DAVID (https://david.ncifcrf.gov/).

## Supplementary Materials

The following are available online at https://www.mdpi.com/article/10.3390/jpm11111068/s1, Table S1: The results of iTRAQ-nano-HPLC-MS/MS analyses of nuclear proteins.
